# Un-“ESCRT”-ed Budding

**DOI:** 10.3390/v3010026

**Published:** 2011-01-18

**Authors:** Mark Yondola, Carol Carter

**Affiliations:** 1 Department of Microbiology, Mount Sinai School of Medicine, New York, NY 10128, USA; E-Mail: mark.yondola@mssm.edu; 2 Department of Molecular Genetics and Microbiology, Stony Brook University, Stony Brook, NY 11794, USA

**Keywords:** influenza virus, M2, ESCRT, budding, HA, NA, cholesterol, membrane rafts

## Abstract

In their recent publication, Rossman *et al.* [1] describe how the inherent budding capability of its M2 protein allows influenza A virus to bypass recruitment of the cellular ESCRT machinery enlisted by several other enveloped RNA and DNA viruses, including HIV, Ebola, rabies, herpes simplex type 1 and hepatitis B. Studies from the same laboratory [2] and other laboratories [3–6] indicate that budding of plasmid-derived virus-like particles can be mediated by the influenza virus hemagglutinin and neuraminidase proteins in the absence of M2. These events are also independent of canonical ESCRT components [2,7]. Understanding how intrinsic properties of these influenza virus proteins permit ESCRT-independent budding expands our understanding of the budding process itself.

## 1. ESCRT-Dependent Budding

Budding is an integral part of virus infection where the objective is exit of a protected viral genome from the infected cells. By necessity, enveloped viruses use host-derived membrane to form the encapsidating membrane for this purpose. Since nucleic acid and lipid do not have intrinsic affinity for each other, the encapsidation process requires proteins to serve as adaptors, as initiators of membrane deformation and as mediators of bud scission. The notion that the ESCRT (*e*ndosomal *s*orting *c*omplex *r*equired for *t*ransport; reviewed in [8,9]) machinery is an active participant in this process came from identification of Tsg101, a component of ESCRT-1, as a binding partner of the budding determinant (*i.e.*, the Pro-Thr-Ala-Pro “Late domain” motif) in the Gag polyprotein of HIV-1 [10–12]. This motif is required for budding of infectious virus particles or plasmid-derived virus-like particles (VLPs). Subsequently, the Late domain motifs P(P)PPY and LYPXnL were found to partner with ESCRT machinery adaptors, namely members of the Nedd4 family of E3 ubiquitin ligases and Alix, respectively (reviewed in [13–15]).

The topology of enveloped virus budding is similar to that of vesicle budding into the lumen of the late endosome/multivesicular body (LE/MVB) that occurs as part of endocytic trafficking. The ESCRT machinery consists of four ESCRT factors (ESCRT-0, -1, -2 and 3) and a regulating ATPase, Vps4 [8,9,16]. During endocytic trafficking, monoubiquitinated cellular cargo encounters ESCRT-0 on the cytoplasmic face of the early endosomal membrane. This molecular interaction initiates sequential recruitment of ESCRT-1, ESCRT-2 and ESCRT-3. In the last step, Vps4 disassembles the complex, permitting recycling of the ESCRT components into the cytosol. In contrast, use of the ESCRT machinery by viruses has been found to be remarkably versatile: e.g., rhabdoviruses use the machinery while alphaviruses do not [17]. Some members of the paramyxovirus family use the machinery while budding of other members appears to be ESCRT-independent [18]. Interestingly, the influenza virus M1 protein binds the ESCRT-1 factor Vps28 but the significance of this interaction remains to be determined since depletion of endogenous Vps28 has no detectable effect on influenza virus budding [19]. In this regard, it should be noted that the cellular Rab11 pathway has been implicated as an alternative to the ESCRT system for both the influenza and respiratory syncytial viruses [20,21]. All retroviruses use ESCRT factors but family members differ based on whether they recruit ESCRT-1, -2, both or neither to facilitate budding events [22–24]; reviewed in [25]. All retroviruses recruit ESCRT-3 to mediate the final membrane fission event. Thus, viruses exploit the ESCRT machinery in a highly selective manner.

## 2. ESCRT-Independent, M2-Mediated Budding

By identifying M2 as the mediator of membrane scission, Rossman *et al.* [1] provide the prototype for an “un-ESCRT-ed” budding mechanism. In the context of the infected cell where all of the viral genes are expressed, assembly and release of influenza virus occurs on plasma membrane rafts (reviewed in [26]). Localization to rafts is driven by the virus-encoded transmembrane proteins, hemagglutinin (HA) and neuraminidase (NA), which have inherent affinity for the high cholesterol, liquid-ordered nature of this membrane microdomain [27]. Protein-protein interaction with HA brings the viral matrix (M1) protein into the budding assemblage [26]. M1 recruits the viral ribonucleoprotein complexes and the non-raft associated transmembrane protein M2 into the emerging bud. Rossman and co-authors [1] propose that M2 protein alone can initiate membrane deformation, bud neck formation and bud neck scission and support this hypothesis by demonstrating that (i) expression of M2 alone in 293T cells resulted in release of enveloped M2-containing particles into tissue culture media and (ii) reconstitution of giant unilamellar vesicles (GUVs) with purified M2 protein alone induced the accumulation of M2-containing vesicles in the lumen of the GUVs. Moreover, they show that these events can be mediated by a synthetic peptide containing the sequence of the 17-amino acid amphipathic helix located in the cholesterol-binding C-terminal cytoplasmic tail of the M2 protein. This sequence includes a known cholesterol interaction motif, termed a “CRAC” motif (*c*holesterol *r*ecognition/interaction *a*mino acid *c*onsensus motif) that is present in several viruses [27]. Mutations introduced into the amphipathic helix reduced budding of M2 from the plasma membrane and GUVs. However, the fact that M2 does not associate with rafts suggests that its intrinsic cholesterol binding property is insufficient to drive budding outside of the context of the *in vitro* assay. Based on their observation that M2 accumulated at the boundary between regions of high and low cholesterol in the GUVs, Rossman *et al.* [1] hypothesize that, in the context of viral replication where all of the viral proteins are expressed, M2 accumulates at the boundary between the HA and NA in raft-like lipid microdomains of high cholesterol content and the M1-ribonucleoprotein complexes in the adjacent plasma membrane. The resulting phase separation in the region between the different membrane microenvironments is suggested to induce a line tension (*i.e.*, a departure from the basal energy level) that promotes bud neck constriction to minimize the strain at the phase boundary. If large enough, this force can drive spontaneous membrane deformation, bud formation, bud neck thinning and, ultimately, bud scission [28]. Overall, this model is very attractive and satisfyingly supported by the experimental findings. Although Rossman *et al.* demonstrate that mutation of the amphipathic helix residues significantly reduces viral titers in cell culture experiments using the Udorn strain of influenza virus [29], mutation of residues within the helix had no effect on virus growth in cell culture experiments using the WSN strain [30]. The explanation for this discrepancy may lie in the fact that two of the three mutations generated by Stewart *et al.* [30] do not lie on the hydrophobic side of the amphipathic helix. It would be of interest to determine the effect of changing bulky hydrophobic residues to alanine within the amphipathic helix in the WSN background. Alternatively, the discrepancy might reflect the fact that the Udorn virus has a filamentous morphology while WSN is spherical. Also, we noted no apparent homology between the influenza type A and type B M2 cytoplasmic tails and, moreover, the type B residues that align with the type A amphipathic helix are not predicted to form a similar structure. It will be interesting to determine whether the influenza type B viruses bud using a similar ESCRT-independent mechanism. Like M2, ESCRT-1 and -2 localize to the neck of budding retroviral particles [31]. Whether assisted by the ESCRT-3 complex or the M2 protein, a fundamental condition for success in bud scission may be the induction of line tension. The inherent line tension-generating property of the raft/non-raft boundary may explain why influenza virus, HIV-1 and other viruses utilize lipid rafts as their budding platform [32].

## 3. Budding Directed by Other Influenza Virus Proteins

Influenza type A virus is unique in having several virus-encoded proteins that are capable of budding on their own. The HA/NA complex, M1 and some isoforms of the NA can also drive particle release when expressed in the absence of any other viral proteins in a plasmid-based system that generates spherical VLPs [2,3–6]. The efficiency of M2-mediated budding relative to HA/NA-mediated budding is very low [2]. While the co-expression of HA/NA and M2 does generate a boost in HA/NA VLP yield, this boost is very modest [2]. This result suggests that other factors are capable of contributing to the scission event. Indeed, Gomez-Puertas *et al.* [4] showed earlier that the M1 protein, when expressed alone, assembles into virus-like budding particles, which are released in the culture medium. In their studies, M1 was the only viral component essential for VLP formation. Latham *et al.* [5] investigated the minimal number of structural proteins necessary for VLP assembly and release using single-gene baculovirus recombinants that expressed in Sf9 cells either the HA, NA, M1 or the M2 proteins of influenza virus A/Udorn/72 (H3N2). Expression of M1 protein alone led to the release of VLPs. Wang *et al.* [33] found that native M2 but not mutant M2 lacking the cytoplasmic tail, effectively targeted M1 to the plasma membrane and produced extracellular M1 VLPs. Interestingly however, these investigators observed that M2 did not bud efficiently as compared with M1 plus M2. It is important to note that these observations are based on studies in the VLP background. Whether M2 alone is necessary for the budding of influenza virus has not yet been determined. M2 certainly contributes to virus budding, as apparent by the 5-log decrease in titer that Rossman obtained using their M2-mutated virus. Whether all of the decrease is attributed to budding deficiency is not known. While the VLP system is very useful to determine which viral proteins contain the necessary determinants of budding and provides the benefit of assessing their relative efficiency in this process, studies in the context of the virus must ultimately be used to determine the interplay between the proteins. Perhaps the budding properties observed for M2, M1, HA and NA reflect a redundancy of this critical function, analogous to retroviral primary and secondary Late domain determinants.

VLPs produced by HA/NA or NA alone exhibit very similar morphology [3,6]. Side-by-side comparisons of this morphology with that of M2-derived VLPs have not yet been performed. HA/NA and NA budding have yet to be examined in the GUVs budding assay to determine whether, like M2, these proteins can mediate all the membrane events required for budding. Interference with Vps4 function by siRNA-mediated depletion or co-expression of influenza virus with dominant-negative (DN) interfering mutants of Vps4 did not inhibit virus budding [19,34]. Similarly, release of budding-competent NA isoforms exhibited resistance to disruption of Vps4 function [7]. Thus, like M2 and the intact influenza virus, NA budding is not inhibited by disruption of the ESCRT machinery through DN Vps4 expression.

Certainly, the results obtained by Rossman *et al.* [1] raise many exciting questions that will stimulate investigations to elucidate the underlying mechanism of M2’s contribution to budding. It will also be very interesting to determine if functional equivalents of M2 are encoded in other virus families budding by ESCRT-independent mechanisms.
